# Incremental value of electroanatomical mapping during pentaspline pulsed field pulmonary vein isolation: the MAP-PFA pilot randomized trial

**DOI:** 10.1093/europace/euag222

**Published:** 2026-08-19

**Authors:** Ahmed S Yassin, Bakhtawar Khan Mahmoodi, Bart A Mulder, Celina V Malyar, Michiel Rienstra, Sing-Chien Yap, Yuri Blaauw

**Affiliations:** Department of Cardiology, University of Groningen, University Medical Center Groningen, Hanzeplein 1, Groningen 9713 GZ, The Netherlands; Department of Cardiology, Thorax Center, Cardiovascular Institute, Erasmus MC, Rotterdam 3015 GD, The Netherlands; Department of Cardiology, University of Groningen, University Medical Center Groningen, Hanzeplein 1, Groningen 9713 GZ, The Netherlands; Department of Cardiology, Thorax Center, Cardiovascular Institute, Erasmus MC, Rotterdam 3015 GD, The Netherlands; Department of Cardiology, University of Groningen, University Medical Center Groningen, Hanzeplein 1, Groningen 9713 GZ, The Netherlands; Department of Cardiology, Thorax Center, Cardiovascular Institute, Erasmus MC, Rotterdam 3015 GD, The Netherlands; Department of Cardiology, University of Groningen, University Medical Center Groningen, Hanzeplein 1, Groningen 9713 GZ, The Netherlands

**Keywords:** Atrial fibrillation, Pulsed field ablation, Electroanatomical mapping, Pulmonary vein isolation, Eccentricity index, Pentaspline catheter

## Abstract

**ClinicalTrials.gov registration:** NCT07014423.

## Introduction

Electroanatomical (EA) mapping enables real-time visualization and precise lesion delivery during atrial fibrillation (AF) ablation.^1^ Its value in single-shot pulsed field ablation (PFA) remains unclear. Given the rapid uptake of PFA and its incremental per-procedure cost, clarifying the value of EA guidance is timely and relevant.^2^ We hypothesized that mapping-guided PFA would produce more central antral lesions, fewer additional applications, and better flower-configuration spline overlap, without prolonging procedure time.

## Methods

This pilot, prospective, randomized, open-label, blinded endpoint (PROBE) trial recruited patients between September and December 2025 at two Dutch centres. Eligible patients (>18 years, documented AF, planned *de novo* PFA PVI) were randomized 1:1 to the blinded group (fluoroscopy/ICE only; EA mapping data collected but blinded from the operator) or the EA-mapping group (additional EA-mapping guidance). The second-generation pentaspline catheter (Farawave NAV, Boston Scientific) was used in both groups. The study complied with the Declaration of Helsinki.

### Study endpoints

**Figure 1:**
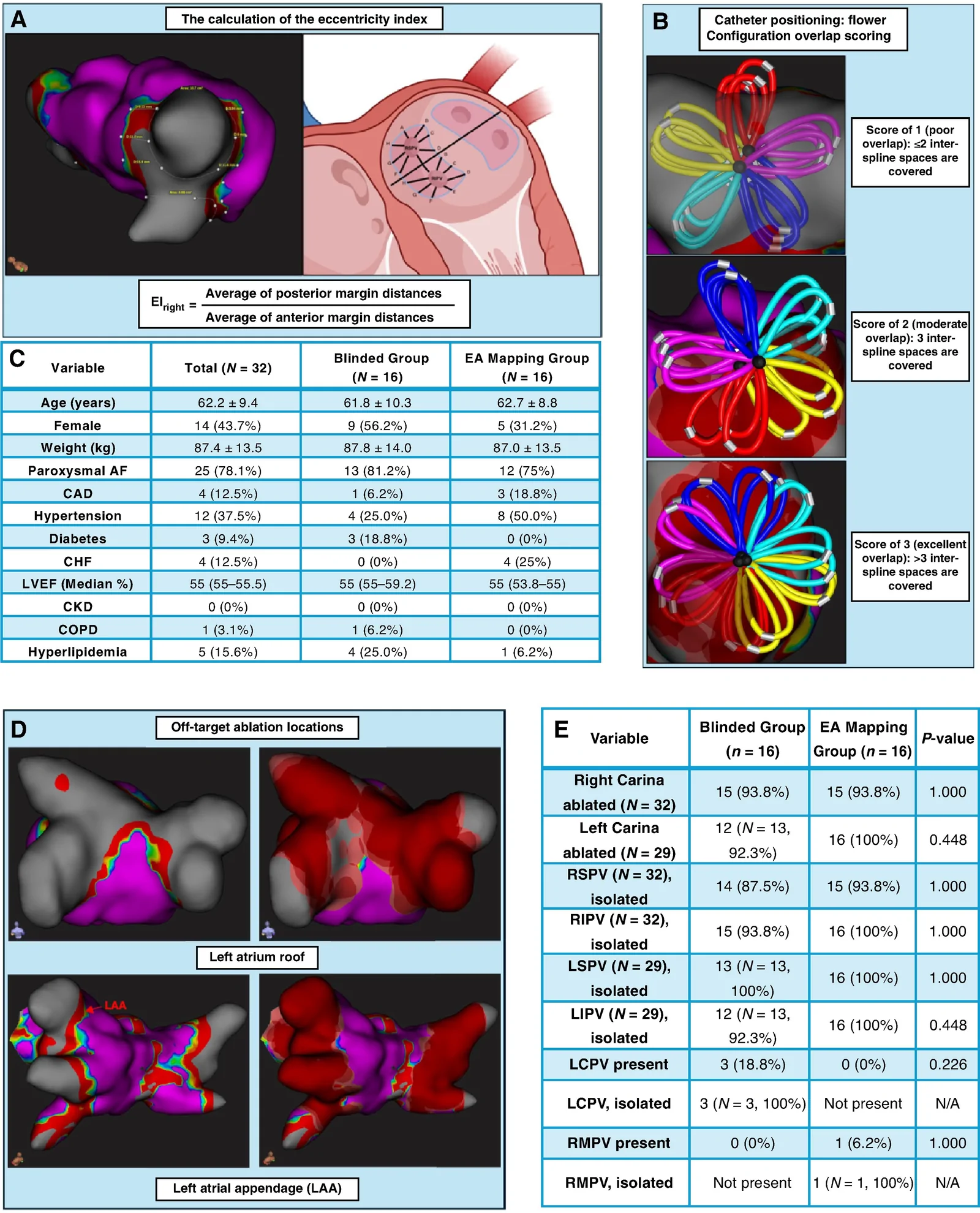
(*A*) Calculation of the eccentricity index (EI). The left panel shows a left atrial (LA) voltage map with annotated anterior and posterior margin distances for the right pulmonary veins (RPVs). The right panel is a schematic drawing of the LA illustrating the same measurements. The equation is displayed within the figure. (*B*) Representative examples of flower overlap configurations and their corresponding quality scores. (*C*) Baseline characteristics. (*D*) Off-target ablation locations. Top: left atrial roof line (left) and corresponding ablation lesion markers (right). Bottom: left atrial appendage with non-ablated LIPV (left) and corresponding ablation lesion markers (right). (*E*) Procedural characteristics. Values without ‘(*n* = xx)’ indicate that all patients in that group were included (total *N* = 32, Blinded group *n* = 16, EA mapping group *n* = 16). Values with ‘(*n* = xx)’ indicate the actual number of patients with available data for that specific variable. EI, eccentricity index. AF, atrial fibrillation; CAD, coronary artery disease; CHF, congestive heart failure; LVEF, left ventricular ejection fraction; CKD, chronic kidney disease; COPD, chronic obstructive pulmonary disease; LAA, left atrial appendage; SD, standard deviation. IQR, interquartile range; PV, pulmonary vein; LIPV, left inferior pulmonary vein; LSPV, left superior pulmonary vein; RIPV, right inferior pulmonary vein; RSPV, right superior pulmonary vein; LCPV, left common pulmonary vein; RMPV, right middle pulmonary vein; SA, surface area.

The primary endpoint was the eccentricity index (average posterior/anterior antral lesion’s margin distances; perfect centricity = 1; >1 = posteriorly eccentric, <1 = anteriorly eccentric; *Figure 1A*),^3^ averaged from right PVs. Although originally described for cryoballoon, this metric is equally applicable to single-shot PFA, as it quantifies the spatial symmetry of the final contiguous ablation lesion relative to the PV ostium/antrum. Left PVs were excluded due to the left atrial appendage (LAA) ridge and anatomical variants. Secondary (exploratory) endpoints: additional touch-up applications (extra PV antrum/carina ablations; post-ablation high-density mapping); procedure time; off-target ablations; incomplete PVIs; and flower-configuration spline-overlap quality (scale 1–3, higher = better), graded by four blinded electrophysiologists (*Figure 1B*).

### Ablation procedure

Post-transseptal puncture, each PV received ≥10 applications (four basket, four flower, and two olive). After initial PFA set, a high-density map (INTELLAMAP ORION™ catheter) followed. Additional ablation was at the operator’s discretion.

### Post-ablation analysis

High-density mapping voltage thresholds were >0.3 mV (normal tissue) and <0.1 mV (scar).^4^ If a right PV was deeply ablated/lacked an antral lesion; eccentricity index was derived from the other right PV (*N* = 6, 18.75%). One patient (EA-mapping group) was excluded from primary analysis (LA posterior wall scarring).

### Statistical analysis

For sample size calculation, assumed 0.2 mean eccentricity index difference (mean = 0.9 in the EA-mapping group, standard deviation (SD) = 0.2 in both groups, α = 0.05, power 80%, and 1:1 randomization), we required 16 per arm. Continuous variables were reported as mean ± SD or median (IQR). Normally distributed variables (Shapiro–Wilk test) were compared using *t* test; non-normally distributed variables using Mann–Whitney *U* test. Categorical variables were reported as frequencies/percentages and compared using Fisher’s exact test. Sample size assumed normality, but non-normal eccentricity index necessitated non-parametric primary analysis. Missing data were excluded per variable. All tests were two-sided, *P* < 0.05 considered significant. Analyses were conducted using R4.4.3.

## Results

A total of 32 patients were enrolled. Baseline characteristics in *Figure 1C*. Median eccentricity index was 0.91 (0.75–1.27) in the EA-mapping group and 1.23 (0.93–1.62) in the blinded group (95% CI (0.02–0.63); *P* = 0.039). A sensitivity analysis on log-transformed eccentricity index confirmed the primary finding (median log(Eccentricity Index) −0.094 vs. + 0.199; 95%CI (0.02–0.61); *P* = 0.039). Total applications were 44 (42–49) in the blinded group vs. 44 (42–46) in the EA-mapping group (*P* = 0.606); additional touch-ups occurred in 3/16 patients (18.8%) vs. 1/16 (6.2%) (*P* = 0.599), with per-patient distributions of: 30, 17, and 8 applications vs. 16 (sum 55 vs. 16). Median procedure time was 57 (53–70) minutes in the blinded group vs. 59 (54–63) minutes in the EA-mapping group (*P* = 0.82); and fluoroscopy time was 10.5 ± 4.6 (*n* = 15) minutes vs. 10.5 ± 2.9 (*n* = 16) minutes (*P* = 0.994). Two off-target ablations (LAA, LA roof) occurred only in the blinded group (*P* = 0.484; *Figure 1D*). Four incomplete PVIs were observed in the blinded group vs. one in the EA-mapping group (*P* = 0.197). Flower-overlap analysis included 65 veins in the EA-mapping group (64 PVs + one RMPV) and 60 veins in the blinded group (three LCPV and one missed LIPV). Median flower-overlap quality score was numerically higher in the EA-mapping group (3 (2.3–3) vs. 2.5 (2–3), *P* = 0.050). Additional results in *Figure 1E*.

## Discussion

In the present study, mapping–guided PFA produced more centrally-located antral lesions than fluoroscopy/ICE–guided PFA. Although not statistically significant, mapping guidance showed numerical improvements in spline overlap (flower-configuration), off-target ablations, incomplete PVIs, and touch-up applications, without prolonging procedure time.

Single-shot fluoroscopy/ICE-guided PVI relies on indirect indicators (wire position, tactile feedback, catheter shape, echocardiography) to infer catheter location,^5^ making reliable central placement challenging. EA mapping overcomes this by allowing visual confirmation of antral location before energy delivery. Yagishita *et al.*^6^ found incomplete antral lesions in 16% of fluoroscopy-only PFA, with no improvement with operator experience, highlighting limitations of indirect visualization. Our study observed incomplete PVI more often in the blinded group, suggesting mapping could mitigate this. Fewer off-target ablations in the EA-mapping group resonates with reports that fluoroscopic guidance alone can result in unintended energy delivery outside the target.^7^

The reduced number of touch-up applications suggest upfront mapping guidance probably yield a complete initial lesion-set, potentially lowering total energy delivery and collateral exposure. The identical median total procedure applications reflect the protocol-mandated minimum. EA mapping did not prolong procedure time, consistent with non-randomized comparisons.^8,9^ The NAVIGATE-PF phase-2 study reported durable isolation in 95% of veins at 60 days with the mapping-integrated catheter.^10^ Our acute-phase study complements these findings by showing that mapping could improve lesion centricity and potentially enhance flower-overlap, plausible prerequisites for durability. Whether these acute improvements translate into superior clinical outcomes remains unknown.

### Limitations

The sample size was small, powered for a mechanistic endpoint, not clinical outcomes, and lacks statistical power to detect secondary outcomes; therefore, the study should be interpreted with caution and as a hypothesis-generating pilot study. The clinical relevance of improved lesion centricity remains unproven, as downstream outcomes (PVI durability/arrhythmia recurrence) were not assessed. Procedure complications/safety endpoints/follow-up were not assessed. LCPV only in the blinded group may have influenced secondary outcomes. Operators unblinding to group-assignment may introduce performance bias.

## Conclusion

In this pilot study, EA mapping-guided PFA resulted in more centrally located antral lesions and numerical improvement in the flower-spline overlap. Further studies are needed to evaluate long-term efficacy.

## Data Availability

Data are available upon reasonable request from the corresponding author.
